# Assessing Reliability in Flywheel Squat Performance: The Role of Sex and Inertial Load

**DOI:** 10.3390/jfmk11010004

**Published:** 2025-12-24

**Authors:** Priscila Torrado, Michel Marina, Jorge Salse-Batán

**Affiliations:** Research Group in Physical Activity and Health (GRAFAiS), Institut Nacional d’Educació Física de Catalunya (INEFC), Universitat de Barcelona (UB), 08038 Barcelona, Spain; jorgesalseb@gmail.com

**Keywords:** half-squat, eccentric, consistency, repeatability, measurement error

## Abstract

**Objectives**: We examined the effects of sex and inertia on within-session reliability of flywheel half-squat performance outcomes. **Methods**: A total of 21 males and 25 females (aged 24.9 and 23.6, respectively) performed two sets of six valid repetitions using four inertial loads. Mean force, mean and peak power, impulse, and work were recorded during concentric and eccentric phases. Intraclass correlation coefficient (ICC), coefficient of variation (CV), typical error (TE), smallest worthwhile change (SWC), and minimal detectable change were calculated. A three-way repeated measures ANOVA was used to identify systematic differences and interaction effects. **Results**: Regardless of inertia or contraction phase, both males and females demonstrated excellent between-set reliability (ICC > 0.803 in males and superior to 0.946 in females) across all variables. Although males showed slightly higher CV values, CVs were good for all variables (≤9%). Overall, good sensitivity (SWC > TE) was observed in the four inertias, with marginal sensitivity (TE > SWC) more frequently observed for the power-related outcomes. Whereas no interactions between Sex × Set × Inertia were observed among the variables, significant interactions between Inertia × Sex were observed in both contraction phases for power-related variables (eccentric peak power, *p* < 0.001; concentric mean power, *p* = 0.032). **Conclusions**: reliability was excellent across all moments of inertia and contraction phases for both sexes, highlighting the importance of considering inertia configuration and sex differences when profiling performance outcomes.

## 1. Introduction

The ability to perform the squat movement is essential for a broad range of functional activities, such as sitting and lifting, as well as for the execution of numerous sport-specific actions [1]. As a multiple-joint exercise, the traditional squat exercise (i.e., gravitatory) has been commonly used in training regimens designed to improve athletic performance or lower-body muscle hypertrophy [2,3]. An alternative to gravitatory or isokinetic devices is the use of flywheel devices (FDs), which have emerged in the past decades, to be used in a gravity-independent scenario [4,5]. Recent studies have described the benefits of training with FDs in reducing the risk of injury [6], improving neuromuscular performance [7], and increasing muscle volume and force [8]. From a biomechanical perspective, FDs provide an inertia-dependent loading profile that enables high-velocity concentric acceleration followed by the absorption of greater eccentric forces, driven by increased and prolonged mechanical tension and work production [9]. These demands promote efficient force transmission through enhanced cross-bridge dynamics and the contribution of passive elastic elements during muscle lengthening [10]. At the neuromuscular level, the transition between ECC and CON phases elicits the recruitment of high-threshold motor units and elevated neural activation [11,12,13]. Moreover, the eccentric overload induced by FDs has been linked to molecular adaptations, including satellite-cell activation [14] and upregulated protein synthesis signaling in fast-twitch fibers [15]. Because these multi-level responses depend on the magnitude and consistency of the mechanical stimulus applied, accurate monitoring of kinetic variables is fundamental to ensure that performance assessment reflects true physiological demands rather than variability introduced by technique or load selection.

In this context, a critical aspect influencing the mechanical stimulus delivered by FDs is the appropriate selection of inertial load. In contrast to the traditional resistance training devices, where training load is controlled by adjusting the added weight according to the individual’s one-repetition maximum, FDs require selecting the moment of inertia to optimize training responses. Previous research demonstrates that variations in inertial load markedly affect performance variables such as concentric (CON) and eccentric (ECC) power outputs [16,17,18]. Furthermore, other kinetic variables, such as force, impulse, or work, are indirectly modulated by inertia-related changes in movement velocity, with lighter inertias allowing faster muscle actions and heavier inertias resulting in a slower execution of the exercise [19]. In addition, the different technique strategies that an athlete has to halt the rotation during the ECC phase affect the load received, potentially attenuating, maintaining, or increasing it [20]. This, together with the parameter- and exercise-dependent repeatability reported by Bollinger et al. [21], suggests that variability between repetitions may be inherent to flywheel devices, particularly with inexperienced subjects [22]. Given this variability, evaluating the reliability of the measurements becomes essential to ensure that observed changes reflect real differences rather than inconsistencies in execution or measurement error [23].

In the context of performance assessment, retest reliability refers to the consistency of a measure across repeated trials under identical conditions [24]. Ensuring that physical capacity metrics meet acceptable reliability standards is essential for their meaningful interpretation [25] when monitoring changes resulting from training programs or interventions [24]. Numerous studies have focused on the reliability and validity of FDs [26,27,28,29,30] and their related kinetic and kinematic variables [31,32], with investigations typically manipulating inertial load to determine its influence on reliability [17,33]. With regard to the FD squat, very good to excellent reliability has been reported previously for mean power [34], peak power [18,35,36], peak force and impulse [37], and mean and peak velocity [27]. Despite the increasing number of studies addressing the reliability of FDs, female representation remains limited, hindering the ability to determine whether sex differences may exist in these contexts. Research examining reliability in flywheel exercises has been conducted almost exclusively in male [16,18,34,35,36,38,39] or mixed participant groups [17,31,32], leaving uncertainty as to whether sex-specific neuromuscular or biomechanical differences influence the stability of FD-derived kinetic outcomes. It is well known that sex-related differences in anthropometric and physiological characteristics may influence the neuromuscular performance and fatigability [40], which in turn could affect the reliability of the kinetic variables obtained during repeated sets of multi-joint exercises. Consistent with this, previous research has shown that males tend to exhibit greater and earlier performance declines across repeated sets, including larger reductions in power outputs and more pronounced decrements in work and impulse compared with females, even after adjusting for body mass [41]. Recent research suggests that sex-related differences in neuromuscular strategies during barbell back squat may influence the stability of performance measures [42]. Van den Tillaar et al. [42] reported that females not only exhibited greater maximal angular hip extension velocity in the final repetitions of 6- and 10-RM, but also demonstrated a different EMG pattern compared to males. The intra-set differences in neuromuscular strategies could increase kinetic output variability, potentially affecting reliability when comparing different sets. Furthermore, the choice of inertia used for testing can alter force and power outputs, with higher inertia affecting males and females differently, as mechanical and neuromuscular responses to increasing loads may vary between sexes [19]. Recent evidence with gravitational loading indicates that load magnitude influences barbell squat kinematics differently across sexes [43], suggesting that the reliability of the kinetic variables in flywheel squats may depend on distinct modulation strategies adopted by males and females as inertia is increased. Such evidence highlights the suitability of examining force, power, impulse, and work when exploring sex-specific reliability patterns. Although previous studies have examined these variables, to the best of our knowledge, sex-specific analyses in FD have been restricted to single-joint [17,19] or upper-body exercises using a single inertial load [44], providing insufficient evidence regarding their behavior in more complex lower-body multi-joint exercises.

To our knowledge, no study examined sex differences in the within-session reliability of CON and ECC kinetic variables during a high-demand exercise such as the FD half-squat. Moreover, it remains unclear whether changes in inertial load affect reliability in a similar or different manner in males and females. Addressing this question is essential for optimizing testing protocols, guiding individualized load prescription, and informing sex-specific monitoring strategies in both research and applied settings. Therefore, the aim of this study was to investigate sex differences in the within-session reliability of concentric and eccentric kinetic performance variables during the flywheel half-squat across a range of inertial loads ranging from 0.06 to 0.13 kg·m^2^.

We hypothesized that (1) kinetic variables would differ across inertial loads and between sexes, and (2) females, despite generally exhibiting lower absolute performance than males, would exhibit similar within-session reliability when repeating sets of the FD half squat.

## 2. Materials and Methods

### 2.1. Experimental Approach

**Figure 1:**
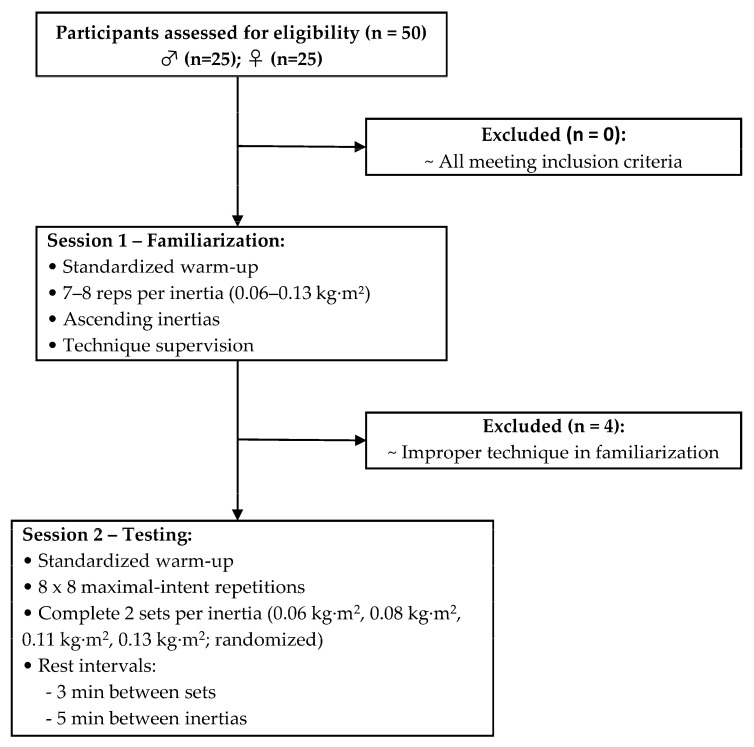
Diagram of the testing procedure. ♂ = Males, ♀ = Females.

This study applied an intra-day repeated-measures design to explore sex differences in the within-session reliability of CON and ECC kinetic variables across four inertial loads. Within this design, physically active sport science students completed a single testing session under a standardized laboratory protocol to assess the intra-day reliability of mean force, mean and peak power, impulse, and work (see Figure 1).

### 2.2. Participants

Fifty physically active sports science students volunteered to participate in this study. A convenience-based sample was recruited for the present study. The sample size was estimated using the calculator proposed by Arifin [45] for reliability studies. The estimation revealed that a sample of 18 subjects would provide an 80% chance to achieve a desired ICC of 0.90, with 0.75 set as the minimally acceptable threshold [24], based on six repetitions per participant. However, a larger sample (n = 46; 21 males and 25 females) was recruited to account for potential within-group dropouts.

Participants were eligible for inclusion if they (1) had at least two years of resistance training experience, (2) had no musculoskeletal injuries in the preceding six months, and (3) did not regularly use FDs as part of their training. Participants who did not meet the established criteria were found ineligible (Figure 1). Testing procedures were explained prior to the start of the study, and participants provided written consent after being informed of the purposes and potential risks. The study was approved by the local Institutional Review Board (IRB00003099) in accordance with the Declaration of Helsinki.

### 2.3. Procedures

Each participant attended the laboratory on two occasions, separated by one-week and scheduled at the same time of day. Ten minutes before starting each testing session, participants performed a standardized warm-up including 5 minutes of light running, mobility exercises, dynamic stretching, and one set of 10 repetitions at incremental velocity with the lowest inertia.

**Figure 2:**
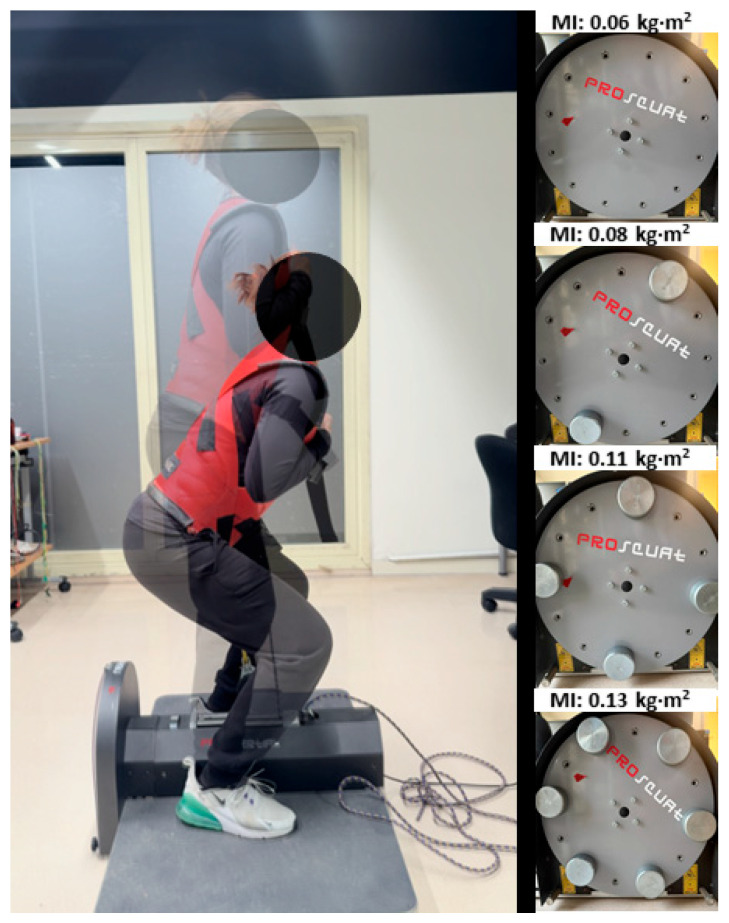
Illustration of the flywheel half-squat protocol. The left panel shows the execution of the exercise across the concentric and eccentric phases. The right panel displays the detailed view of the counterweight arrangement for each moment of inertia (MI) tested in the study (0.06, 0.08, 0.11, 0.13 kg·m^2^).

During the first session, which served as familiarization with the FD ProSquat (Proinertial^®^, Barcelona, Spain), participants performed one set of 7–8 repetitions with each of the four moments of inertia (Figure 2). In this session, the inertias were presented in ascending order, from the lowest to the highest, to allow participants to gradually adapt to the increasing inertial demands. Within each set, participants were instructed to gradually increase the movement velocity, reaching maximal effort during the final three repetitions. All technical aspects of the execution were reviewed and corrected by the same experimenter to ensure a proper performance in the subsequent testing session. Participants who were unable to demonstrate proper technique during the familiarization session were excluded from the study.

The testing session consisted of 8 sets of 8 repetitions of the FD half-squat, with two sets performed per inertial condition (0.06, 0.08, 0.11, and 0.13 kg·m^2^). Moments of inertia were randomized to minimize order effects and avoid systematic bias due to fatigue accumulation on performance outcomes. In order to allow for maintenance of power output, a rest interval of 3 minutes was provided between sets of the same inertia [18], and 5 minutes between different inertial loads. To ensure standardized execution, participants were instructed to perform all repetitions at maximal intended velocity from the very first repetition, keeping their heels in contact with the ground throughout the CON (upward) phase, while progressively braking during the ECC (downward) phase until the knee reached a 90° angle (see Figure 2). Squat depth was visually controlled by the same researcher to ensure a consistent range of motion. Arm position was standardized by crossing them with the hands placed on the opposite shoulders. Stance width and foot position were self-selected within a controlled technical range (approximately shoulder-width) during the familiarization and then kept constant across all sets. An experienced researcher provided standardized verbal instructions and encouragements across participants to ensure consistent technical execution and depth throughout each repetition. To prevent potential bias in effort or execution, participants did not receive any performance-related feedback until the completion of the study. Subjects were required to avoid any form of lower limb training 48 h before the testing session and were instructed to refrain from consuming caffeine at least 3 h prior to testing.

### 2.4. Data Processing

Kinetic measurements of both CON and ECC phases were collected separately using a rotatory axis encoder (Chronojump, Chronojump-Boscosystem^®^, Barcelona, Spain) with an accuracy of ±1 mm and a sampling rate of 1000 Hz. From the raw displacement and time data, mean force (N), mean and peak power (W), impulse (N·s), and work (J) were computed for each contraction phase and used for analysis. Signal was analyzed offline using the Chronojumpsoftware (Chronojump^®^, Barcelona, Spain, v2.3.0). Despite instructions to perform every repetition at maximal velocity from the outset, the first two repetitions were discarded from analysis because they consistently yielded lower outputs. As noted by other studies, the first two repetitions serve to accelerate the FD and stabilize squat amplitude [32]. Consequently, only the remaining six repetitions per set were used for the analysis.

### 2.5. Statistical Analysis

For each variable, between-set reliability was assessed by comparing the average of six valid repetitions from the first and second sets, analyzed separately for each phase. Normal distribution of data was checked by the Shapiro–Wilk’s test, and homogeneity of variance was verified with the Levene’s test.

To assess systematic inter-set differences and the extent to which these differences were influenced by sex and/or inertia, we applied a mixed-model three-way repeated measures ANOVA for both phases separately after confirming the normality of the data. This model included sex (n = 2: male, female) as the between-subject factor, and set (n = 2: S1, S2) and inertia (n = 4: 0.06, 0.08, 0.11, and 0.13 kg·m^2^) as within-subject factors. When the sphericity was not assumed, Greenhouse–Geisser’s corrections were applied. Post hoc comparisons were performed when appropriate using Bonferroni adjustment. The effect size was reported as partial eta squared (ⴄ^2^p), with values of 0.01, 0.06, and 0.14 interpreted as small, medium, and large effects, respectively [46].

Intraclass correlation coefficients (ICC) and coefficients of variation (CV) were calculated to assess relative and absolute reliability, respectively. Following the criteria established by Koo and Li [47], ICC values were categorized as poor (<0.5), moderate (0.5–0.75), good (0.75–0.9), and excellent (>0.9). All ICCs were reported with 95% CI. According to Cormack, et al. [48], values of CV < 5% were considered good, and between 5 and 10% as acceptable. As a complementary indicator of absolute reliability, we reported the typical error (TE), also referred to as the standard error of the measurement (SEM). This metric reflects the precision of the measurement and quantifies the amount of random error inherent to the test, making it more suitable for evaluating absolute reliability in repeated-measures contexts [25]. Smallest worthwhile change (SWC, defined as 0.2 × between-subject SD [24]) was calculated to detect meaningful changes between-sets, and minimal detectable change (MDC = 1.96 × $\sqrt{2}$ × SEM) to determine the smallest amount of change that can be interpreted as a real difference. The MDC was additionally expressed as a percentage (%MDC) by dividing the MDC by the mean of all observations, enhancing comparability across measures and studies [49]. Test usefulness was assessed by comparing the TE with the SWC, where TE < SWC indicates ‘good’, and TE > SWC indicates ‘marginal’ sensitivity [50]. All statistical analyses were performed with PASW 18 software (SPSS Inc., Chicago, IL, USA). The level of significance was set at *p* < 0.05.

## 3. Results

A total of 50 volunteers were initially screened; however, only 46 proceeded to the testing session, as four participants were excluded following the familiarization session for failing to demonstrate adequate execution technique. Table 1 shows the basic descriptive characteristics of the participants, divided by sex.

Between-sets reliability scores for all variables and moments of inertia are detailed for CON and ECC phases in Table 2 and Table 3, respectively. Regardless of inertia, or contraction phase, between-set reliability was excellent across all measured variables. Both males and females demonstrated excellent relative reliability, with ICCs ranging from 0.803 to 0.995 in males and from 0.946 to 0.995 in females.

Absolute reliability, expressed as coefficient of variation (CV%), was consistently good in females, with values ≤ 5% across all conditions, with the exception of ECC peak power. For this variable, CV% values ranged between 5% and 8% during the ECC phase, with the highest variability observed at the lowest inertia (0.06 kg·m^2^) and the lowest variability at the highest inertia (0.13 kg·m^2^). In males, CV% values ranged from 2% to 9% across variables. Notably, for mean force, mean power, and peak power, the CV% in males decreased progressively as the inertia increased.

The SWC values were consistently higher than TE in the CON phase, except for mean power in males at 0.08 and 0.11 kg·m^2^, and for peak power in females at all inertias except 0.13 kg·m^2^. During the ECC phase, the TE in females remained below SWC for most variables, except for peak power at 0.06 kg·m^2^ of inertia. However, in males, the TE exceeded the SWC for mean force at 0.11 kg·m^2^, as well as for peak power across all inertias.

**Figure 3:**
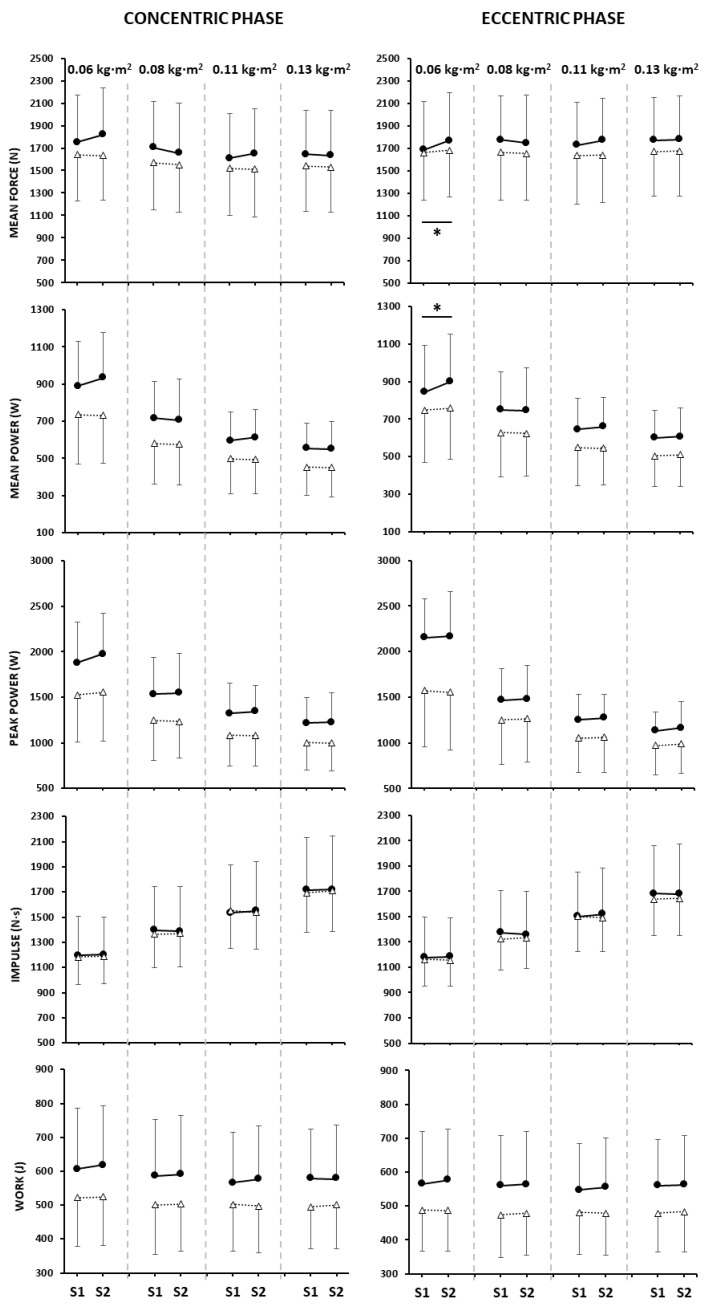
Mean values of set 1 (S1) and set 2 (S2) for each one of the four inertial loads (0.06, 0.08, 0.11, and 0.13 kg·m^2^). Each row corresponds to a different kinetic variable, and columns depict the concentric (**left**) and eccentric (**right**) phases. For each inertia, S1 and S2 values are connected to illustrate inter-set differences. Gray dotted vertical lines separate the four inertial loads within each graph. Data for males (black circles) and females (white triangles) are displayed separately. * Significant post hoc differences in the Inertia × Set interaction.

Figure 3 presents the descriptive analysis (mean and standard deviation) and provides a visual summary of kinetic variables (rows) and contraction phases (columns). Within each panel, males and females are displayed separately, and Set 1 and Set 2 are connected for each inertia to illustrate inter-set differences.

No significant interactions between Sex × Set × Inertia were observed among the studied parameters. However, significant main effects and two-way interactions were found, as detailed below. For mean force, there was a significant inertia per set interaction in the CON phase (F = 3.7, *p* = 0.013, ⴄ^2^p = 0.078). Post hoc analysis indicated that in set 1, force at 0.06 kg·m^2^ was higher than at 0.11 and 0.013 kg·m^2^ (*p* < 0.001), while in set 2, force at 0.06 kg·m^2^ exceeded all other inertias (*p* < 0.001). A main effect of inertia was also observed (F = 20.29, *p* < 0.001, ⴄ^2^p = 0.316), with values being higher at the lowest inertia (0.06 kg·m^2^) compared to the other inertia levels. The Inertia × Set interaction was also observed in the ECC phase (F = 4.57, *p* = 0.007, ⴄ^2^p = 0.094), where between-sets differences were revealed at 0.06 kg·m^2^ (S1 < S2; *p* < 0.001). The main effect of set was also significant (F = 4.11, *p* = 0.049, ⴄ^2^p = 0.085), with lower values in set 1.

Concerning the mean power, a significant interaction between Inertia × Sex was observed in the CON phase (F = 3.03, *p* = 0.032, ⴄ^2^p = 0.064). Post hoc analyses indicated that males produced higher values than females at all moments of inertia (Inertia × Sex: *p* ≤ 0.04), although both sexes showed a similar stepwise decline in mean power as inertia increased from 0.06 to 0.13 kg·m^2^ (*p* ≤ 0.0001). Previous observations are strengthened by a strong main effect of inertia (F = 195.8, *p* < 0.0001, ⴄ^2^p = 0.817) and a main effect of sex (F = 5.21, *p* = 0.01, ⴄ^2^p = 0.141), with males outperforming females overall. In the ECC phase, a significant Inertia × Set interaction was found (F = 3.84, *p* = 0.011, ⴄ^2^p = 0.08), with lower values at set 1 compared to set 2 at 0.06 kg·m^2^ (*p* = 0.001). Significant main effects of inertia (F = 107.39, *p* < 0.0001, ⴄ^2^p = 0.709) and set (F = 4.78, *p* = 0.034, ⴄ^2^p = 0.098) indicated, on one hand, a decline in power with increasing inertia, and, on the other hand, lower values in set 1 compared to set 2.

For peak power, both the CON and ECC phases showed main effects of sex (CON: F = 7.25, *p* = 0.01, ⴄ^2^p = 0.141; ECC: F = 7.42, *p* < 0.009, ⴄ^2^p = 0.144), and inertia (CON: F = 129.25, *p* < 0.0001, ⴄ^2^p = 0.746; ECC: F = 151.95, *p* < 0.0001, ⴄ^2^p = 0.775), with values declining stepwise as inertia increased from 0.06 to 0.13 kg·m^2^ (*p* ≤ 0.004). In addition, a significant interaction between Inertia × Sex was detected in the ECC phase (F = 12.09, *p* < 0.0001, ⴄ^2^p = 0.216), with males showing higher values at 0.06 and 0.11 kg·m^2^ (*p* ≤ 0.043).

Impulse showed consistent outcomes for both contraction phases. A main effect of inertia was observed (F ≥ 593.62, *p* < 0.0001, ⴄ^2^p ≥ 0.931), with impulse values increasing stepwise as inertia increased (*p* < 0.0001) in the CON and ECC as well. For the work parameter, a main effect of inertia was observed in the CON phase (F = 9.99, *p* < 0.0001, ⴄ^2^p = 0.185), with higher values at 0.06 kg·m^2^ compared with the other inertias (*p* ≤ 0.003). In the ECC phase, only the main effect of sex (F = 4.42, *p* < 0.041, ⴄ^2^p = 0.091) was found for work, with males outperforming females.

## 4. Discussion

The present investigation aimed to examine the effects of varying FD moment of inertia on the FD half-squat, and to determine how these variations influence intra-session reliability for mean force, mean and peak power, impulse, and work, with a specific focus on identifying sex differences in these variables. As expected, males outperformed females significantly, while higher inertia reduced power-related measures and increased impulse in a similar way in both sexes, showing the excellent reliability across contraction phases for both males and females, and good sensitivity in most conditions, with no apparent effect of inertia on reliability, with exceptions in peak power. Because of the lack of similar studies assessing sex differences in reliability, there are scarce comparable values for these results with females.

### 4.1. Influence of Sex on Reliability

Previous research analyzing different inertial loads has reported good inter-session reliability in males during the FD squat exercise [27,35,51], particularly from the second testing session onwards, once familiarization effects had stabilized [18]. However, to date, only one study [34] has examined intra-session reliability in the FD half-squat exercise, reporting excellent reliability for mean power (ICC = 0.87–0.99). Nevertheless, their investigation was conducted exclusively with male participants. Relative to the results reported by Ryan et al. [34], the current study showed excellent reliability in mean power for males (ICC ≥ 0.803; CV ≤ 9%) and females (ICC ≥ 0.946; CV ≤ 8%), in both the CON and ECC phases, indicating high measurement consistency under all conditions (see Table 2 and Table 3). In the present study, CVs for mean force and for mean and peak power were highest in males at the lowest inertia and either remained constant or progressively decreased as the inertial load increased, with the lowest variability observed at the highest load. A similar pattern was observed in females for peak power, where CV values decreased progressively across inertias.

On the other hand, comparisons between TE and the SWC scores provided insight into the sensitivity of the measures beyond their overall consistency [52]. The fact that TE values were consistently below SWC for all variables and conditions in females suggests a good sensitivity with this type of testing setup in this group. The only exceptions observed in females are the peak power, where TE values exceeded SWC at all inertias except 0.13 kg·m^2^ in the CON phase and 0.06 kg·m^2^ in the ECC phase, indicating marginal sensitivity. This indicates that while overall between-sets reliability was excellent for the majority of variables in females, the capacity to detect meaningful changes during the CON phase was limited for peak power, except with the highest inertia. In contrast to females, measurement sensitivity in males was compromised on some occasions for mean and peak power, as TE values exceeded SWC at the intermediate inertias (0.08 and 0.11 kg·m^2^) in the CON phase. Similarly, while peak power showed limited sensitivity across all inertial loads in the ECC phase, marginal sensitivity for mean power was observed, but only at 0.11 kg·m^2^. The previous observations suggest that higher inertial loads in the half-squats enhance the stability of power-related measures. Such findings could be explained by faster CON–ECC transitions at lower inertias, which may compromise timing precision [31]. Our results align with those reported by Beato et al. [35], who observed high reliability but low sensitivity of peak power measurements obtained during flywheel squat in both CON and ECC phases in male participants, when tested with an inertia of 0.061 kg·m^2^.

To ensure accurate estimates of reliability, it is crucial to control for potential sources of systematic error [24]. The inertia × set interaction observed during the ECC phase for mean force and mean power indicated that both sexes produced higher values in the second trial at 0.06 kg·m^2^. However, while the ANOVA revealed no set × sex interaction, indicating similar trends in both sexes, the reliability analysis provided additional insight. While the DIF_set_ observed in males at 0.06 kg·m^2^ exceeded the SWCs for the same measure and the associated TE remained below SWC (Table 3), the DIF_set_ in females was not sufficient to exceed the SWC. This observation implies that, although both groups performed better in the second set with 0.06 kg·m^2^, the magnitude of this difference was only meaningful in males [52]. These sex-related differences are particularly relevant in FD exercises, where the overloaded ECC phase is known to elicit strong neuromuscular potentiation and post-activation performance enhancement [53]. Our findings are consistent with previous studies that reported slight increases in ECC power [18] or even acute performance improvements [54,55], suggesting the involvement of post-activation potentiation enhancement (PAPE) following a single set of flywheel exercise. Influence of PAPE could explain the improvement in males at 0.06 kg·m^2^, as their greater proportion of type II fibers compared with females is more sensitive to PAPE mechanisms [56,57,58]. Given that the underlying mechanism of post-activation potentiation involves the phosphorylation of myosin light chains predominantly in type II muscle fibers, a lower proportion of these fibers is likely to attenuate the potentiation response in females [58]. Supporting this rationale, Seitz and Haff Seitz and Haff [57] reported that strength status modulates the PAPE response. Participants with higher absolute strength reached peak performance after a single conditioning set, whereas those with lower strength required multiple sets. Given that females generally exhibit lower absolute strength than males [59], as also confirmed in our study, a single set may fall below the minimum effective dose required to elicit a meaningful potentiation in females. However, the absence of consistent potentiation across our variables suggests that the observed pattern cannot be fully explained by PAPE. The higher TE observed during the CON phase at 0.06 kg·m^2^ may reflect greater variability during the acceleration phase, and could be associated with technical familiarization at this inertia, potentially influencing the subsequent ECC phase. These observations reinforce the importance of complementing traditional statistical analyses with sensitivity metrics to determine whether systematic differences between trials represent trivial variation or meaningful change [24,25].

In the present study, typical error values for mean force and for peak and mean power consistently declined as inertia increased, particularly in the CON phase, reinforcing the notion that heavier loads promote more stable measurements. On the other hand, impulse and work exhibited the lowest variability across inertial conditions, with CVs consistently below 5% in both sexes, with a TE that consistently fell below the SWC. These findings suggest that impulse and work are reliable and highly stable variables. Nevertheless, two drawbacks affect the interpretation of impulse and work: (1) the absence of significant sex interactions for DIF_set_ reinforces the notion that fluctuations in these variables likely reflect random variability rather than true performance changes; and (2) the fact that DIF_set_ did not exceed SWC, suggests that both variables lack sufficient sensitivity to detect acute performance improvements or subtle neuromuscular adaptations within a single session.

On the other hand, contrary to the findings of Spudić et al. [44], who observed greater measurement error in females for force and power-related measures when exerting low-rowing, our result revealed that males exhibited greater CV% and TE than females. Such disparities reinforce the relevance of considering sex as a factor in performance analysis [60].

### 4.2. Influence of Inertial Settings

Earlier studies have primarily investigated how the inertial setup influences performance outcomes. However, despite these consistent findings regarding load-dependent mechanical outputs, research directly comparing males’ and females’ responses to the inertial setup is scarce. To date, the studies that have reported sex-specific values have focused exclusively on single-joint tasks, such as the leg extension [19] and the leg curl exercise [17]. The present study is the first to examine the effects of different inertial loads on performance outcomes during the squat exercise with an FD while reporting male and female results separately. Males showed higher performance-related values than females in mean and peak power during the CON phase, and peak power and work in the ECC phase. Our results align with previous authors [61], who observed higher force and power values in males, certainly due to a greater muscle mass.

It is well known that lower inertial loads (e.g., 0.025 kg·m^2^) elicit higher concentric peak power [18], whereas higher moments of inertia (e.g., 0.075–0.1 kg·m^2^) result in greater force production and work, but at lower movement velocities and power outputs [19,62]. Contrary to the common assumption that greater flywheel inertia consistently enhances CON force [19,20], our participants exhibited the highest mean CON force and work at the lowest inertia (0.06 kg·m^2^), with no pairwise differences among the higher inertias. A plausible explanation is a strategy shift at higher inertial loads, where the reduced CON velocity and the adoption of a braking strategy to cope with the ensuing ECC demand could alter the within-repetition force–time profile, leading the force outputs of moderate and high inertias to converge to a similar level. However, this may also be related to incomplete familiarization with higher loads. Given that work followed a similar pattern, and considering that work is determined by the product of mean force and displacement, it is possible that participants slightly adjusted the range of motion at the higher inertias to compensate for the increased load, in spite of the visual control of the technical execution.

Notably, for CON mean power and ECC peak power, our results revealed that, although males outperformed females, both sexes exhibited a similar progressive decline in performance as inertia increased. These results are consistent with previous studies using FD with different inertial settings, which have shown that males exhibit higher absolute values than females [17,19], and confirm that greater resistance favors higher impulse and force production at the expense of power, as previously reported under gravity-dependent loads [63]. According to Carroll et al. [37], as inertial load increases, an enhanced impulse may reflect a reduction in the unweighting phase and a concomitant increase in time under mechanical tension. From a neuromuscular and molecular standpoint, this prolonged tension at higher inertias may elicit distinct patterns of motor unit fatigue and metabolic stress, both of which are recognized as key stimuli for activating molecular signaling pathways associated with muscle hypertrophy [15]. However, it is very important to note that gravity-dependent loading is influenced by the absolute mass moved, magnifying sex-based differences due to generally higher muscle mass in males. In contrast, inertial loading is proportional to the force generated by the individual [64], thus potentially attenuating sex differences in impulse, as performance depends more on the fore-time application pattern than on absolute force capacity.

This study presents limitations that should be acknowledged. First, the sample consisted of sport science students with resistance training experience, which may limit the generalizability of the findings to other populations. Second, had knee and hip flexion–extension been monitored using goniometers or a 3D motion-capture system instead of relying on visual observation of the experimenter, it is plausible that a greater degree of variability in squat depth would have been identified. Finally, the menstrual cycle phase of female participants was not controlled, which may have introduced additional variability in neuromuscular performance.

## 5. Practical Applications

The excellent within-session reliability observed for kinetic variables such as mean force, power, impulse, and work indicates that these parameters can be confidently used by coaches to monitor performance during FDs’ half-squat exercises performed in conditioning training sessions. The absence of sex-related differences in reliability further supports the use of these parameters for both males and females under comparable testing conditions. In practice, the tendency to diminish measurement variability with higher inertial loads highlights the importance of selecting appropriate moments of inertia to enhance the stability of power-related metrics. Moreover, the higher variability observed at the lowest inertia suggests that additional familiarization sessions should improve control and technical precision at these loads.

## 6. Conclusions

Our findings indicate an excellent within-session reliability of concentric and eccentric kinetic variables during flywheel half-squat performance across all inertial loads in both sexes. Although females showed slightly lower measurement error and greater consistency than males, these sex differences did not compromise the overall robustness of the reliability estimates. Overall, these results demonstrate that flywheel-derived kinetic variables can be reliably assessed within a single session in both males and females, reinforcing their suitability for monitoring acute neuromuscular performance in trained individuals.

Additionally, the potentiation observed at lower inertial loads for force and power during the eccentric contraction phase highlights the importance of considering inertia configuration and potential sex-related differences when interpreting acute performance outcomes.

## Figures and Tables

**Table 1:** Descriptive characteristics of the participants.

	N	Age (Years)	Mass (kg)	Height (cm)	Body Mass Index (kg/m^2^)	Training (Day/Week)	Strength Training Experience (Years)
Males	21	24.9 (5.1)	76.6 (9.9)	180.5 (5.3)	23.4 (2.7)	4.3 (1.3)	9.9 (6.7)
Females	25	23.6 (6)	62.7 (7.7)	165.1 (4)	22.8 (2.8)	4.4 (1.2)	10.1 (4.1)

N, number of participants of each group; data are presented as mean (standard deviation).

**Table 2:** Inter-set reliability components for females (F) and males (M) during the concentric phase (CON) of the different moments of inertia.

MI	SEX	DIFset	ICC (95% CI)	CV%	SWC	TE	MDC	MDC%	TE vs. SWC
MEAN FORCE									
0.06	F	60 ± 51	0.991 (0.978–0.996)	2%	97.0	46.0	127.6	8	good
0.08	F	55 ± 61	0.991 (0.979–0.996)	3%	95.9	45.5	126.1	8	good
0.11	F	52 ± 42	0.994 (0.985–0.997)	2%	93.6	36.3	100.5	7	good
0.13	F	53 ± 41	0.993 (0.984–0.997)	3%	91.4	38.2	106.0	7	good
0.06	M	120 ± 118	0.964 (0.910–0.985)	5%	106.1	100.6	278.9	16	good
0.08	M	111 ± 127	0.962 (0.907–0.985)	5%	113.4	110.5	306.3	19	good
0.11	M	102 ± 72	0.977 (0.944–0.991)	4%	104.6	79.4	220.0	14	good
0.13	M	58 ± 45	0.991 (0.979–0.996)	3%	109.7	52.1	144.3	9	good
MEAN POWER									
0.06	F	47 ± 45	0.984 (0.964–0.993)	4%	65.8	41.6	115.4	16	good
0.08	F	29 ± 31	0.990 (0.978–0.996)	4%	62.4	31.2	86.5	15	good
0.11	F	25 ± 22	0.992 (0.981–0.996)	4%	51.9	23.2	64.4	13	good
0.13	F	25 ± 18	0.990 (0.977–0.996)	4%	47.7	23.8	66.1	15	good
0.06	M	78 ± 64	0.964 (0.910–0.985)	7%	68.5	65.0	180.1	22	good
0.08	M	63 ± 53	0.959 (0.898–0.983)	7%	71.8	72.7	201.6	32	marginal
0.11	M	54 ± 48	0.943 (0.858–0.977)	6%	55.5	66.2	183.6	34	marginal
0.13	M	31 ± 23	0.981 (0.954–0.992)	4%	55.8	38.4	106.5	21	good
PEAK POWER									
0.06	F	104 ± 88	0.983 (0.963–0.993)	5%	165.6	177.8	299.2	19	marginal
0.08	F	93 ± 84	0.977 (0.948–0.990)	5%	117.3	134.4	246.5	20	marginal
0.11	F	58 ± 46	0.988 (0.972–0.995)	4%	114.7	137.9	174.2	16	marginal
0.13	F	71 ± 51	0.978 (0.951–0.990)	5%	86.0	74.1	172.8	17	good
0.06	M	161 ± 135	0.954 (0.886–0.981)	6%	165.8	107.9	492.8	29	good
0.08	M	123 ± 95	0.964 (0.910–0.985)	6%	141.7	88.9	372.5	27	good
0.11	M	125 ± 111	0.920 (0.804–0.968)	7%	97.5	62.8	382.3	32	good
0.13	M	73 ± 47	0.979 (0.948–0.991)	5%	99.9	62.3	205.4	19	good
IMPULSE									
0.06	F	35 ± 39	0.988 (0.973–0.995)	2%	50.2	27.5	76.2	6	good
0.08	F	33 ± 28	0.994 (0.986–0.997)	2%	53.2	20.6	57.2	4	good
0.11	F	34 ± 25	0.995 (0.989–0.998)	2%	51.9	18.4	50.9	3	good
0.13	F	51 ± 30	0.993 (0.983–0.997)	2%	56.6	23.7	65.6	4	good
0.06	M	33 ± 22	0.994 (0.986–0.998)	2%	61.2	23.7	65.7	6	good
0.08	M	59 ± 87	0.976 (0.941–0.990)	3%	63.1	48.9	135.5	10	good
0.11	M	38 ± 52	0.994 (0.984–0.997)	2%	63.5	24.6	68.2	4	good
0.13	M	42 ± 31	0.994 (0.986–0.998)	2%	69.6	27.0	74.8	4	good
WORK									
0.06	F	28 ± 32	0.977 (0.948–0.990)	4%	30.3	23.0	63.8	12	good
0.08	F	22 ± 15	0.991 (0.980–0.996)	3%	35.7	17.0	47.0	9	good
0.11	F	19 ± 12	0.993 (0.984–0.997)	3%	27.6	11.6	32.0	6	good
0.13	F	26 ± 18	0.984 (0.964–0.993)	4%	31.0	19.6	54.3	11	good
0.06	M	31 ± 19	0.990 (0.976–0.996)	4%	34.4	17.2	47.7	8	good
0.08	M	42 ± 38	0.971 (0.929–0.988)	5%	41.6	35.4	98.2	18	good
0.11	M	28 ± 32	0.981 (0.952–0.992)	4%	31.3	21.6	59.7	11	good
0.13	M	25 ± 20	0.988 (0.971–0.995)	3%	36.3	19.9	55.1	10	good

MI: moment of inertia (kg·m^2^); DIF: absolute difference between set 1 and set 2; ICC (95% CI): ICC measure and 95% confidence interval; TE: typical error; CV%: coefficient of variation; SWC: smallest worthwhile change; MDC: minimal detectable change; MDC%: minimal detectable change in percentage.

**Table 3:** Inter-set reliability components for females (F) and males (M) during the contraction eccentric phase (ECC) of the different moments of inertia.

MI	SEX	DIF	ICC (95% CI)	CV%	SWC	TE	MDC	MDC%	TE vs. SWC
MEAN FORCE									
0.06	F	55 ± 45	0.993 (0.985–0.997)	2%	84.2	35.2	97.7	6	good
0.08	F	64 ± 59	0.989 (0.975–0.995)	3%	81.9	42.9	119.0	7	good
0.11	F	63 ± 55	0.990 (0.978–0.996)	3%	83.8	41.9	116.2	7	good
0.13	F	49 ± 42	0.993 (0.985–0.997)	2%	84.2	35.2	97.7	6	good
0.06	M	92 ± 82	0.988 (0.971–0.995)	4%	85.4	46.8	129.6	8	good
0.08	M	102 ± 113	0.964 (0.912–0.986)	4%	83.4	79.2	219.4	13	good
0.11	M	96 ± 61	0.980 (0.950–0.992)	4%	81.6	57.7	160.0	9	good
0.13	M	62 ± 47	0.989 (0.974–0.996)	3%	85.4	44.8	124.1	7	good
MEAN POWER									
0.06	F	47 ± 46	0.985 (0.967–0.994)	4%	55.1	33.8	93.6	12	good
0.08	F	36 ± 31	0.989 (0.976–0.995)	4%	52.8	27.7	76.8	12	good
0.11	F	28 ± 25	0.991 (0.980–0.996)	4%	46.3	22.0	60.9	11	good
0.13	F	27 ± 21	0.990 (0.976–0.995)	4%	43.8	21.9	60.7	12	good
0.06	M	62 ± 45	0.988 (0.972–0.995)	6%	50.6	27.7	76.7	9	good
0.08	M	59 ± 52	0.964 (0.910–0.985)	6%	48.6	46.1	127.8	19	good
0.11	M	54 ± 44	0.952 (0.881–0.980)	6%	42.9	47.0	130.3	22	marginal
0.13	M	31 ± 26	0.981 (0.954–0.992)	4%	41.8	28.8	79.9	14	good
PEAK POWER									
0.06	F	196 ± 200	0.946 (0.877–0.976)	8%	124.4	144.6	400.7	26	marginal
0.08	F	103 ± 103	0.976 (0.946–0.989)	6%	105.5	81.7	226.5	18	good
0.11	F	90 ± 68	0.977 (0.949–0.990)	6%	96.0	72.8	201.8	19	good
0.13	F	65 ± 45	0.985 (0.966–0.993)	5%	84.3	51.6	143.1	15	good
0.06	M	253 ± 269	0.803 (0.515–0.920)	9%	91.9	203.9	565.2	26	marginal
0.08	M	145 ± 101	0.933 (0.835–0.973)	7%	89.7	116.1	321.8	24	marginal
0.11	M	134 ± 138	0.855 (0.642–0.941)	7%	71.4	135.9	376.8	33	marginal
0.13	M	95 ± 76	0.940 (0.853–0.976)	6%	84.0	102.9	285.2	27	marginal
IMPULSE									
0.06	F	34 ± 32	0.989 (0.975–0.995)	2%	41.5	21.8	60.4	5	good
0.08	F	28 ± 21	0.995 (0.989–0.998)	2%	43.9	15.5	43.1	3	good
0.11	F	34 ± 19	0.995 (0.989–0.998)	2%	48.6	17.2	47.7	3	good
0.13	F	46 ± 26	0.992 (0.982–0.996)	2%	53.2	23.8	65.9	4	good
0.06	M	32 ± 23	0.994 (0.986–0.998)	2%	62.6	24.2	67.2	6	good
0.08	M	64 ± 107	0.965 (0.914–0.986)	3%	60.8	56.9	157.6	12	good
0.11	M	37 ± 43	0.994 (0.984–0.997)	2%	67.2	26.0	72.2	5	good
0.13	M	37 ± 28	0.995 (0.987–0.998)	2%	70.4	24.9	69.0	4	good
WORK									
0.06	F	25 ± 30	0.973 (0.938–0.988)	4%	24.1	19.8	54.9	11	good
0.08	F	20 ± 13	0.991 (0.980–0.996)	3%	28.9	13.7	38.0	8	good
0.11	F	18 ± 11	0.993 (0.983–0.997)	3%	24.9	10.4	28.9	6	good
0.13	F	25 ± 17	0.983 (0.962–0.993)	4%	28.5	18.6	51.6	11	good
0.06	M	26 ± 17	0.991 (0.977- 0.996)	4%	30.5	14.4	40.0	7	good
0.08	M	38 ± 33	0.971 (0.927–0.988)	5%	35.4	30.2	83.6	16	good
0.11	M	26 ± 29	0.981 (0.953–0.992)	3%	30.5	21.0	58.3	11	good
0.13	M	23 ± 19	0.988 (0.971–0.995)	3%	34.0	18.6	51.7	10	good

MI: moment of inertia (kg·m^2^); DIF: absolute difference between set 1 and set 2; ICC (95% CI): ICC measure and 95% confidence interval; TE: typical error; CV%: coefficient of variation; SWC: smallest worthwhile change; MDC: minimal detectable change; MDC%: minimal detectable change in percentage.

## Data Availability

The data supporting the findings of this study are available from the corresponding author (priscilatorradopineda@gmail.com) upon a reasonable request.

## Abbreviations

The following abbreviations are used in this manuscript:

FD	Flywheel device
CON	Concentric
ECC	Eccentric
ICC	Intraclass correlation coefficient
CV	Coefficient of variation
SEM	Standard error of the measurement
SWC	Smallest worthwhile change
MDC	Minimal detectable change
TE	Typical error
MI	Moment of inertia
DIF_set_	Difference between sets
PAPE	Post-activation potentiation enhancement
